# AMP-activated protein kinase-dependent nuclear localization of glyceraldehyde 3-phosphate dehydrogenase in senescent human diploid fibroblasts

**DOI:** 10.18632/aging.203825

**Published:** 2022-01-12

**Authors:** Jee Young Sohn, Hyeok-Jin Kwak, Ji Heon Rhim, Eui-Ju Yeo

**Affiliations:** 1 Department of Medicine, College of Medicine, Gachon University, Incheon 21999, Republic of Korea; 2 Department of Health Sciences and Technology, GAIHST, Gachon University, Incheon 21999, Republic of Korea; 3 Bio-New Material Development, NineBioPharm Co., Ltd., Cheongju 28161, Republic of Korea; 4 Department of Biochemistry, College of Medicine, Gachon University, Incheon 21999, Republic of Korea

**Keywords:** glyceraldehyde-3-phosphate dehydrogenase, AMP-activated protein kinase, nuclear localization, senescence, human diploid fibroblasts

## Abstract

Glyceraldehyde-3-phosphate dehydrogenase (GAPDH) is a key glycolytic enzyme that participates in various cellular events, such as DNA repair and apoptosis. The functional diversity of GAPDH depends on its intracellular localization. Because AMP-activated protein kinase (AMPK) regulates the nuclear translocation of GAPDH in young cells and AMPK activity significantly increases during aging, we investigated whether altered AMPK activity is involved in the nuclear localization of GAPDH in senescent cells. Age-dependent nuclear translocation of GAPDH was confirmed by confocal laser scanning microscopy in human diploid fibroblasts (HDFs) and by immunohistochemical analysis in aged rat skin cells. Senescence-induced nuclear localization was reversed by lysophosphatidic acid but not by platelet-derived growth factor. The extracellular matrix from young cells also induced the nuclear export of GAPDH in senescent HDFs. An activator of AMPK, 5-Aminoimidazole-4-carboxamide-1-β-D-ribofuranoside (AICAR), increased the level of nuclear GAPDH, whereas an inhibitor of AMPK, Compound C, decreased the level of nuclear GAPDH in senescent HDFs. Transfection with AMPKα siRNA prevented nuclear translocation of GAPDH in senescent HDFs. The stimulatory effect of AICAR and serum depletion on GAPDH nuclear translocation was reduced in AMPKα1/α2-knockout mouse embryonic fibroblasts. Overall, increased AMPK activity may play a role in the senescence-associated nuclear translocation of GAPDH.

## INTRODUCTION

Cellular senescence is a complex and heterogeneous process characterized by cell cycle arrest after a limited number of cell divisions. Telomeres shorten with each mitotic division in cells, and a DNA damage response terminally arrests the cell cycle as a protective mechanism [1]. The gradual accumulation of senescent cells during aging can be associated with a progressive decline in physiological functions, promoting tissue damage and making proinflammatory environments favorable for various age-associated diseases, such as cancer, diabetes, cardiovascular diseases, and neurodegenerative diseases [2–4]. Because the incidence of age-related diseases is expected to rise as the global population of elderly individuals over 60 years is increasing rapidly [5], promising therapies and strategies for the prevention and treatment of age-related disorders are urgently needed [6].

Among the events associated with cellular senescence, changes in the expression and secretion of extracellular matrix (ECM) components, such as collagens, glycoproteins, proteoglycans, and ECM-associated proteins, and ECM remodeling enzymes such as matrix metalloproteinases are included [7]. Although cellular senescence is generally thought to be an absolute and irreversible process, the possibility of reversing age-related decline in regenerative properties by exposure to a young systemic environment has been suggested [8]. In addition, young ECM has been shown to induce young phenotypes in senescent human diploid fibroblasts (HDFs) [9]. Similarly, young and adult ECM improve cardiac function, whereas aged ECM accelerates the aging phenotype in cardiomyocytes [10], supporting the utilization of ECM-based cardiac regenerative medicines [11].

Glyceraldehyde-3-phosphate dehydrogenase (GAPDH) is a key glycolytic enzyme that catalyzes the conversion of glyceraldehyde-3-phosphate into 1,3-biphosphoglycerate, producing ATP and pyruvate through anaerobic glycolysis in the cytoplasm. GAPDH also participates in various cellular events, such as DNA replication and repair, transcriptional and post-transcriptional regulation of gene expression, membrane fusion and transport, autophagy, apoptosis and cellular senescence [12–16]. The functional diversity of GAPDH depends on its intracellular localization to the nucleus and other subcellular organelles such as polysomes, the ER, and the Golgi [17]. Previous studies have shown that GAPDH is translocated to the nucleus upon glucose starvation, serum depletion, oxidative stress, and genotoxic stress, through post-translational modifications of GAPDH, such as phosphorylation, oxidation, acetylation, glycosylation, S-nitrosylation, S-glutathionylation, and poly ADP-ribosylation [12, 13, 15, 18–23], and its binding with other molecules, such as Siah1 and SIRT1 [15, 24–27]. Several independent studies have shown the role of nuclear GAPDH in the pathogenesis of several age-related diseases. For instance, under oxidative conditions, GAPDH is oxidized and translocated to the nucleus to form aggregates, which act as a seed to accelerate amyloidogenesis, resulting in neurodegenerative diseases, including Alzheimer's and Parkinson’s diseases [22, 28].

AMP-activated protein kinase (AMPK) is a serine/threonine protein kinase consisting of a catalytic subunit α and regulatory subunits β and γ. AMPK mainly functions as an energy sensor that regulates cellular and organismal metabolism in eukaryotes [29–33]. When energy (ATP) production decreases and the ratio of AMP to ATP increases in cells, AMPK is activated by binding of AMP or ADP to its γ subunit and induces the phosphorylation of the α subunit at Thr^172^ via upstream AMPK kinases, such as liver kinase B1 (LKB1) and Ca^2+^-calmodulin-dependent protein kinase kinase (CaMKK) β. In contrast, AMPK activity is inhibited by dephosphorylation at Thr^172^ by protein phosphatases 2A/2C and phosphorylation at Ser^485/491^ of the α_1_/α_2_ subunits by other kinases, such as Akt, protein kinase A, and ERK [34]. AMPK coordinates energy metabolism with other cellular processes, such as cell growth, mitochondrial homeostasis, endoplasmic reticulum stress, autophagy, and apoptosis by orchestrating various signaling cascades, including mTOR and SIRT1 [34–37]. AMPK activation can occur in response to other stressful and pathological conditions, such as hypoxia, hyperosmosis, muscle contraction, production of reactive oxygen species (ROS), and exposure to genotoxic drugs [33, 38, 39].

Additionally, basal levels of protein or AMPK activity increase in senescent cells and aged tissues [4, 40, 41]. AMPK may play protective roles in aging and age-associated diseases, presumably by reducing ROS, fibrosis, and immunosuppression of critical cells or organs [42–45]. Aging is also associated with a decline in AMPK activation in response to various insults [41, 46]. In either case, AMPK has been considered a potential therapeutic target for metabolic diseases, including type 2 diabetes, as well as other age-associated diseases [33, 47–49]. Because previous studies have revealed that AMPK is an important regulator of the GAPDH nuclear translocation in young HDFs [50], we hypothesized that the increased AMPK activity with senescence may change the intracellular localization of GAPDH, which might play a critical role in age-related functional decline and diseases. Therefore, we investigated the possibility of reversing the senescent changes in GAPDH by modifying the AMPK activity and ECM.

## RESULTS

### Nuclear localization of GAPDH in senescent HDFs and skin cells of aged rats

**Figure 1:**
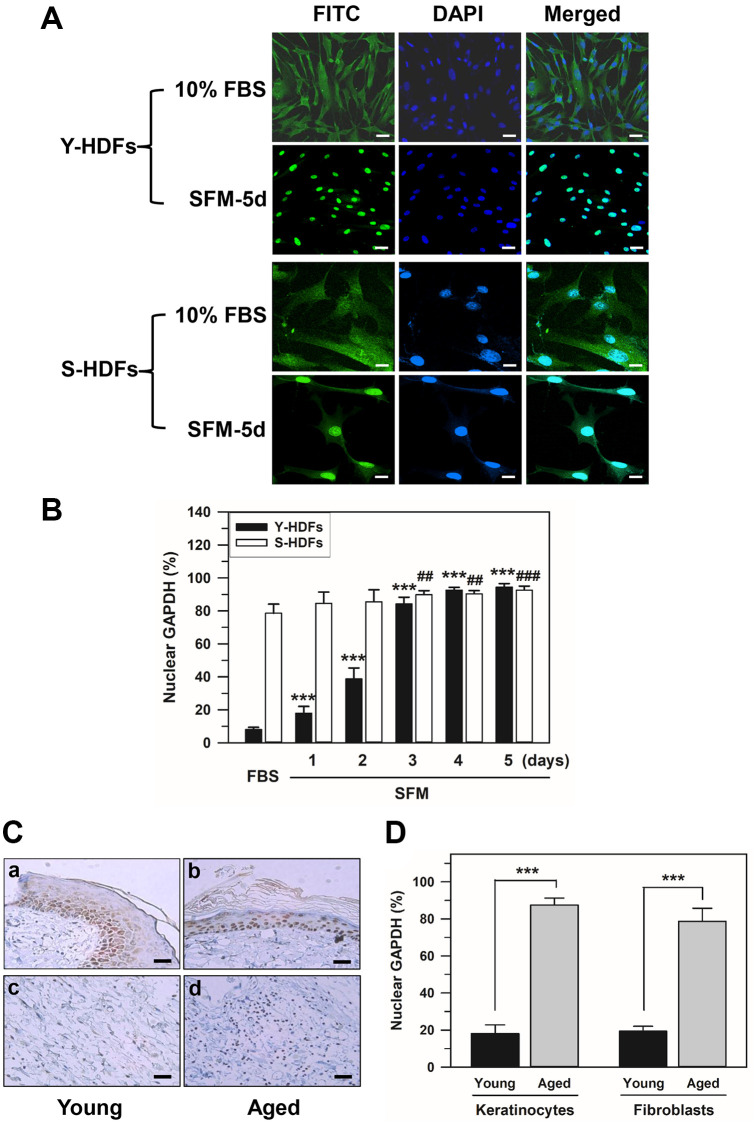
**Nuclear accumulation of GAPDH in senescent HDFs.** (**A** and **B**) Young (Y-HDFs, PD 16) and senescent (S-HDFs, PD 72) cells were maintained in DMEM containing 10% FBS for 2 days and then serum-depleted by incubation with SFM for the indicated times (1–5 days). Cells were immunostained with monoclonal anti-GAPDH antibody and FITC-conjugated anti-mouse secondary antibodies, and analyzed by confocal laser scanning microscopy (magnification, 100×; scale bar, 50 μm). Immunostained young and senescent cells with serum (10% FBS) and without serum for 5 days (SFM-5d) are shown in A. The number of young and senescent HDFs having nuclear GAPDH with or without cytosolic GAPDH was counted, and the percentage distribution was calculated (*n* = 10 for total replicates) and plotted as means ± standard deviations in B. ^***^p < 0.001 (Y-HDFs), ^##^*p* < 0.01 and ^###^*p* < 0.001 (S-HDFs), compared with 10% FBS-treated control cells. (**C**) The levels of GAPDH in the back skin cells from young (6 months, a and c) and aged (24 months, b and d) rats were detected by immunohistochemistry and the epithelial layers containing mainly keratinocytes (a and b) and fibroblasts (c and d) were photographed by light microscopy and the ×200 magnified photos with 50 μm scale bar are shown. Each experiment was performed at least three times with similar results. (**D**) The number of keratinocytes and fibroblasts with nuclear GAPDH was counted in young and aged skin (**C**), the percentage of cells with nuclear GAPDH was calculated (*n* = 8 for total replicates) and plotted as means ± standard deviations. ^***^p < 0.001, compared between young and aged skin.

We investigated the intracellular distribution of GAPDH in young and senescent HDFs with population doubling (PD) of 16 and 72, respectively, by confocal laser scanning microscopy of immunostained HDFs. Confocal images of young and senescent cells (Y-HDFs and S-HDFs, respectively) cultured in 10% fetal bovine serum (FBS) medium or in serum-free medium for 5 days (SFM-5d) are shown in Figure 1A. In young cells, similar to a previous study [50], GAPDH was mainly located in the cytosol with 10% FBS, and the serum withdrawal (SFM) resulted in GAPDH relocation into the nucleus. However, in senescent cells, levels of nuclear GAPDH far exceeded those of cytosolic GAPDH even in 10% FBS medium, and cytosolic GAPDH was further relocated into the nucleus by serum depletion. GAPDH migration to the nucleus upon serum depletion was confirmed by staining the nucleus with DAPI. Confocal images for GAPDH are shown alongside DAPI-stained and merged images in Figure 1A.

The number of cells with nuclear GAPDH with or without cytosolic GAPDH (nuclear +/− cytosol) was counted in the confocal images. The percentage distributions were compared in young and senescent cells cultured in 10% FBS medium for 2 days followed by serum depletion for 5 consecutive days (Figure 1B). Statistically significant differences between groups were determined by one-way ANOVA (*F*(5,54) = 1032.474, *p* < 10^–3^) followed by Dunnett’s T3 post hoc test. In young cells, compared to 8.05% in 10% FBS medium, serum depletion resulted in a gradual increase (^***^p < 0.001) in the portion of nuclear GAPDH from day 1 until day 5 (17.95% on day 1, 38.74% on day 2, 84.19% on day 3, 92.63% on day 4, and 94.44% on day 5). Moreover, a significant (^##^*p* < 0.01) shift of cytosolic GAPDH into the nucleus was observed from days 3 to 5 of serum depletion in senescent cells with both cytosolic and nuclear GAPDH. The percentage of nuclear GAPDH dominant senescent cells was 84.56% on day 1, 85.52% on day 2, 89.85% on day 3, 90.40% on day 4, and 92.61% on day 5 in SFM medium, compared to 78.58% in 10% FBS medium.

Although cellular senescence contributes to aging and age-related diseases, the mechanisms are poorly understood [51]. To confirm that GAPDH is localized in the nucleus of aged or senescent cells of living organisms, young and aged rat skin was prepared, and GAPDH distribution was examined by immunohistochemistry. The distribution of GAPDH in the back skin cells of 6-month-old (young) and 24-month-old (aged) rats showed similar results to the *in vitro* results (Figure 1C). When immunostained with monoclonal anti-GAPDH antibody, the majority of skin cells, both keratinocytes and fibroblasts, showed predominant cytosolic GAPDH distribution in young rats, whereas the majority of these skin cells showed predominant nuclear distribution in aged rats. The number of keratinocytes and fibroblasts with nuclear GAPDH was counted in young and aged skin. The percentage of cells with nuclear GAPDH was calculated and plotted in Figure 1D. Statistically significant differences between young and aged skin were determined by one-way ANOVA (*F*(3,28) = 486.941, *p* < 10^–3^) followed by Dunnett’s T3 post hoc test. Our data showed that the nuclear localization of GAPDH in the skin cells of aged organisms, keratinocytes and fibroblasts increased significantly (^***^p < 0.001) compared to that in young skin cells.

### Growth factor-dependent relocation of nuclear GAPDH to the cytoplasm

**Figure 2:**
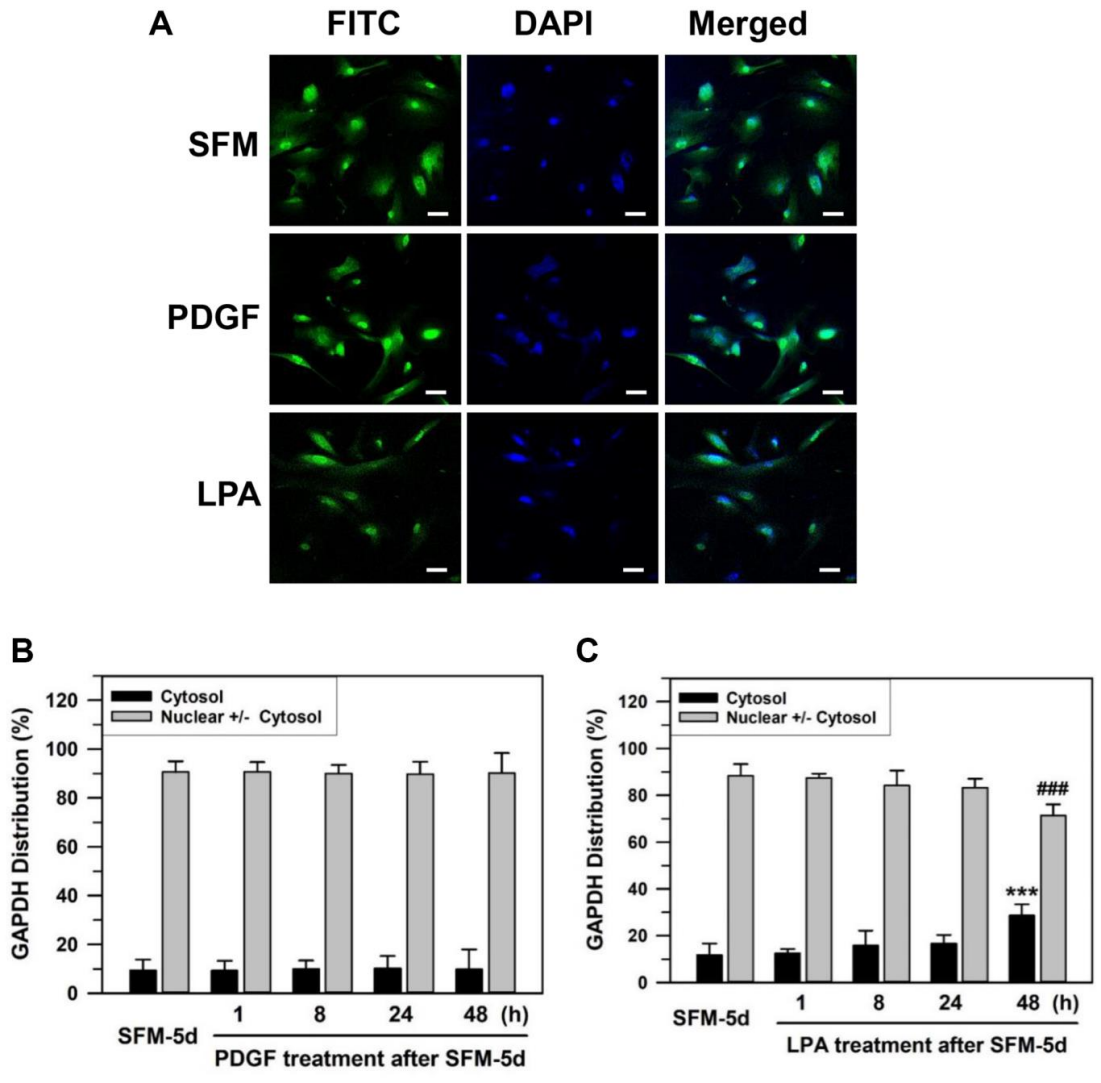
**Effect of growth factor re-addition on the nuclear localization of GAPDH after serum depletion of senescent HDFs.** (**A**) Subconfluent senescent HDFs were serum-depleted by incubation in SFM for 5 days. PDGF (100 ng/ml) or LPA (30 μM) was then added to the serum-depleted cells for 2 days. Cells were immunostained against GAPDH and analyzed by confocal laser scanning microscopy. Immunostained cells with re-addition of PDGF or LPA are shown in A (×100 with 50 μm scale bar). (**B** and **C**) After the re-addition of PDGF (**B**) or LPA (**C**) for the indicated times (1–48 h), the number of cells with cytosolic GAPDH alone (Cytosol) and cells having nuclear GAPDH with or without cytosolic GAPDH (Nuclear +/− Cytosol) was counted, and the percentage distributions were calculated (*n* = 8 for total replicates) and plotted as means ± standard deviations. ^***^p < 0.001 (Cytosol) and ^###^*p* < 0.001 (Nuclear +/− Cytosol), compared with vehicle-treated control SFM-5d cells.

Previously, we have suggested that senescent cells respond to a naturally occurring phospholipid growth factor, lysophosphatidic acid (LPA), which works through G-protein coupled receptors, but not platelet-derived growth factor (PDGF), which works through a receptor tyrosine kinase [52]. To assess the change in GAPDH location upon growth factor re-addition to the media, the effects of LPA and PDGF were compared. Senescent HDFs were deprived of serum by culturing with SFM for 5 days and then grown further in 100 ng/mL PDGF or 30 μM LPA for 1–48 h (Figure 2). GAPDH in senescent HDFs was located mainly in the nucleus after serum deprivation for 5 days. A portion of nuclear GAPDH was exported to the cytoplasm upon LPA addition but not upon PDGF addition to the senescent HDFs (Figure 2A). The statistically significant difference between serum-depleted cells and PDGF- or LPA-treated cells for 1–48 h was determined by one-way ANOVA (*F*(4,35) = 17.543, *p* < 10^–3^) followed by Dunnett’s T3 post hoc test. The addition of PDGF yielded nuclear GAPDH dominant HDFs of 90.69% at 1 h, 90.05% at 8 h, 89.78% at 24 h, and 90.18% at 48 h with *p*-values greater than 0.05, which was not significant for all results when compared to the control SFM-treated for 5 days (90.60%) (Figure 2B). In contrast, LPA addition yielded nuclear GAPDH dominant HDFs of 87.47% at 1 h, 84.21% at 8 h, 83.35% at 24 h and 71.37% at 48 h (^###^*p* < 0.001) when compared to the control SFM-treated for 5 days (88.32%) (Figure 2C). These data suggest that LPA, but not PDGF, can induce the export of nuclear GAPDH in senescent HDFs.

### Effect of ECM on nuclear localization of GAPDH

**Figure 3:**
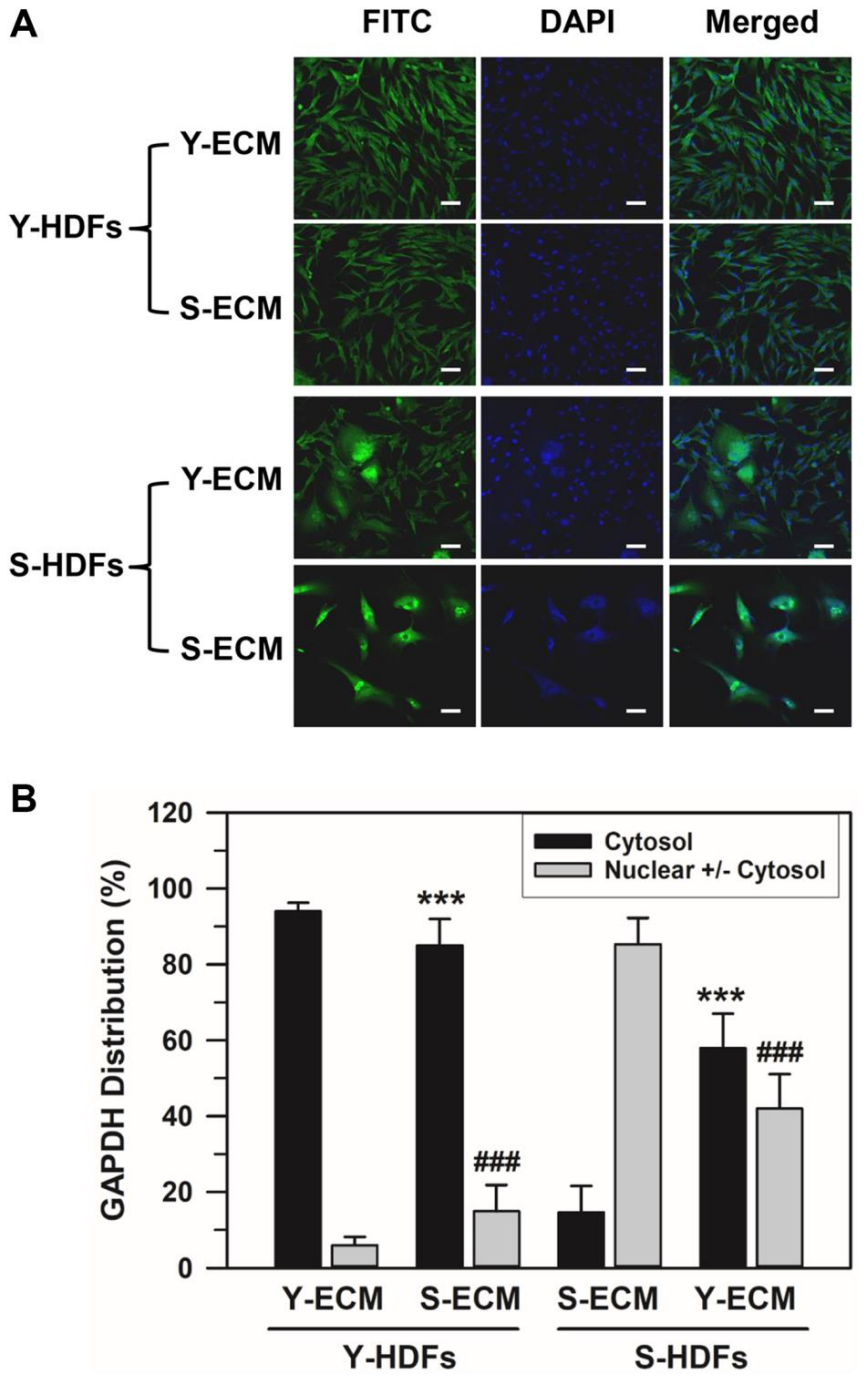
**Effect of young ECM on the nuclear localization of GAPDH in senescent HDFs.** (**A**) Young (Y-HDFs, PD 16) and senescent (S-HDFs, PD 75) cells were seeded onto cover slides coated with young ECM (Y-ECM) or senescent ECM (S-ECM). After incubation for 5 days, cells were immunostained against GAPDH and analyzed by confocal laser scanning microscopy (magnification, 100×; scale bar, 50 μm). (**B**) The number of cells with cytosolic GAPDH alone (Cytosol) and cells having nuclear GAPDH with or without cytosolic GAPDH (Nuclear +/− Cytosol) was counted, and the percentage distributions were calculated (*n* = 18 for total replicates) and plotted as means ± standard deviations. ^***^p < 0.001 (Cytosol) and ^###^*p* < 0.001 (Nuclear +/− Cytosol), compared between Y-ECM and S-ECM in Y-HDFs and S-HDFs.

To determine the effect of young and senescent ECM (Y-ECM and S-ECM, respectively) on the nuclear localization of GAPDH, subconfluent young (Y-HDFs, PD 16) and senescent HDFs (S-HDFs, PD 75) were seeded onto Y-ECM- or S-ECM-coated cover slides in 6-well plates and incubated in SFM for 5 days before immunofluorescence staining. Although the young HDFs did not show significant morphological changes in different ECMs, S-HDFs in Y-ECM showed morphological differences from the regular S-HDFs in S-ECM (Figure 3A). A portion of S-HDFs in Y-ECM showed young-like phenotype. The number of HDFs with cytosolic GAPDH alone and HDFs with nuclear GAPDH with or without cytosolic GAPDH were counted, and the percentage distributions are plotted in Figure 3B. Statistically significant differences between groups were determined by one-way ANOVA (*F*(3,68) = 499.517, *p* < 10^–3^) followed by Dunnett’s T3 post hoc test. As shown in Figure 3B, S-ECM reduced the cytosolic GAPDH levels in Y-HDFs from 94.03 to 85.06% (^***^p < 0.001), and Y-ECM increased those in S-HDFs from 14.68 to 57.98% (^***^p < 0.001). Similarly, S-ECM increased the nuclear GAPDH levels in Y-HDFs from 5.97 to 14.94% (^###^*p* < 0.001) and Y-ECM reduced those in S-HDFs from 85.32 to 42.02% (^###^*p* < 0.001). These data suggest that young ECM induces a young phenotype in a portion of senescent cells and consequently changes the distribution of GAPDH.

### Effect of AMPKα protein and AMPK activity modifiers on GAPDH distribution

**Figure 4:**
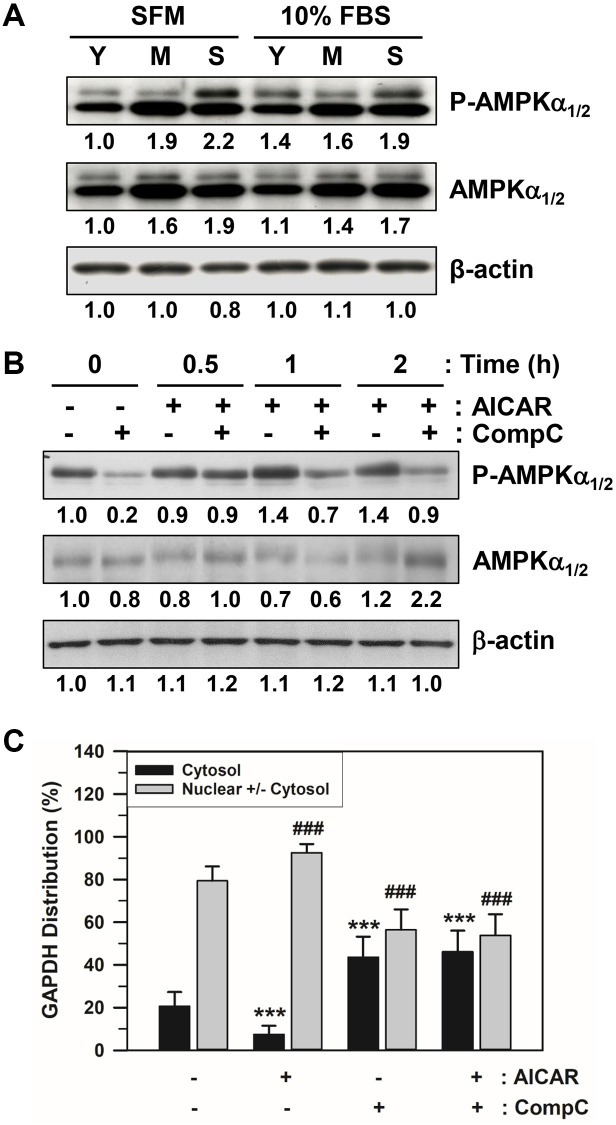
**Effects of AICAR and CompC on AMPK activation and GAPDH distribution in senescent HDFs.** (**A**) Subconfluent young (Y, PD 12), middle (M, PD 48), and senescent (S, PD 86) HDFs were incubated with SFM or 10% FBS medium for 2 days. (**B**) Subconfluent young (PD 20) and senescent (PD 74) HDFs were treated with vehicle (−) or 1 mM AICAR and/or 10 μM CompC (+) for 2 days. Cells in A and B were lysed in a lysis buffer, and 45 μg of protein from each lysate was assessed for the levels of phosphorylated AMPKα1/2 on Thr172 (P-AMPKα1/2), total AMPKα1/2, and β-actin by western blot analysis. The band densities were normalized against β-actin and the fold changes compared to that of young cells (Y/SFM) in A and vehicle treated control cells (−/−) in B are written under each band. (**C**) Subconfluent senescent (PD 72) HDFs were treated with vehicle (−) or 1 mM AICAR and/or 10 μM CompC (+) for 2 days. Cells were immunostained against GAPDH and analyzed by confocal laser scanning microscopy. The number of cells with cytosolic GAPDH alone (Cytosol) and cells having nuclear GAPDH with or without cytosolic GAPDH (Nuclear +/− Cytosol) was counted, and the percentage distributions were calculated (*n* = 40 for total replicates) and plotted as means ± standard deviations. ^***^p < 0.001 (Cytosol) and ^###^*p* < 0.001 (Nuclear +/− Cytosol), compared with vehicle-treated control cells (−/−).

The amount of total AMPKα_1/2_ and phosphorylated AMPKα_1/2_ on Thr^172^ (P-AMPKα_1/2_) in HDFs at different stages (Y: young cells with PD 12, M: middle cells with PD 48, and S: senescent cells with PD 86) were assessed via western blot analysis. The total and phosphorylated AMPKα bands of senescent HDFs after either SFM or 10% FBS addition were darker than the others (Figure 4A and Supplementary Figure 1), indicating that the amount of total activated AMPKα_1/2_ was higher.

Numerous chemical reagents have been utilized *in vivo* and *in vitro* to modulate AMPK activity and AMPK-dependent cellular functions. The cell-permeable nucleoside 5-aminoimidazole-4-carboxamide-1- β-D-ribofuranoside (AICAR) is one of the most commonly used AMPK agonists. In contrast, the cell-permeable pyrazolopyrimidine derivative 6-[4-(2-Piperidin-1-yl-ethoxy)-phenyl]-3-pyridin-4-yl-pyrazolo [1,5-a] pyrimidine (Compound C: abbreviated to CompC) is a potent AMPK blocker via ATP-competitive inhibition. In this study, we used these AMPK activity modifiers. To determine the effects of AICAR and CompC on AMPK activation and GAPDH distribution, subconfluent senescent HDFs were serum-deprived for 2 days and were treated with vehicle, 1 mM AICAR. and/or 10 μM CompC for 2 days. Following treatment with AICAR and CompC, the cells were assessed by western blot analysis. The addition of AICAR to senescent HDFs increased AMPK activity, and the activation was negated by the addition of CompC (Figure 4B and Supplementary Figure 2).

The effects of AICAR and CompC were examined using the number of subconfluent senescent HDFs with GAPDH in the cytosol alone (cytosol) or the nucleus, regardless of cytosolic GAPDH (nuclear +/− cytosol). Following treatment with AICAR and CompC, senescent cells were analyzed by immunofluorescence staining and confocal laser scanning microscopy. The number of cells having cytosolic GAPDH only (cytosol) and cells having nuclear GAPDH with or without cytosolic GAPDH (nuclear +/− cytosol) was counted, and the percentage distributions were plotted (Figure 4C). Statistically significant differences between groups were determined by one-way ANOVA (*F*(3,156) = 220.508, *p* < 10^–3^) followed by Dunnett’s T3 post hoc test. Compared with HDFs without treatment with AICAR or CompC (−/−), the number of HDFs with cytosolic GAPDH only decreased significantly (^***^p < 0.001) from 20.62 to 7.47% with the addition of AICAR, but increased significantly (^***^p < 0.001) to 43.60% with the addition of CompC. As expected, the number of HDFs with nuclear GAPDH with or without cytosolic GAPDH increased significantly (^###^*p* < 0.001) from 79.38 to 92.53% with the addition of AICAR, but decreased to 56.40% with the addition of CompC (^###^*p* < 0.001). When AICAR and CompC were both added to the medium, the number of HDFs with nuclear GAPDH was significantly reduced, similar to when only CompC was added. These data suggest that AMPK activation might be required for the nuclear translocation of GAPDH in senescent HDFs.

### Effects of AMPKα siRNA transfection on the nuclear accumulation of GAPDH in senescent HDFs

**Figure 5:**
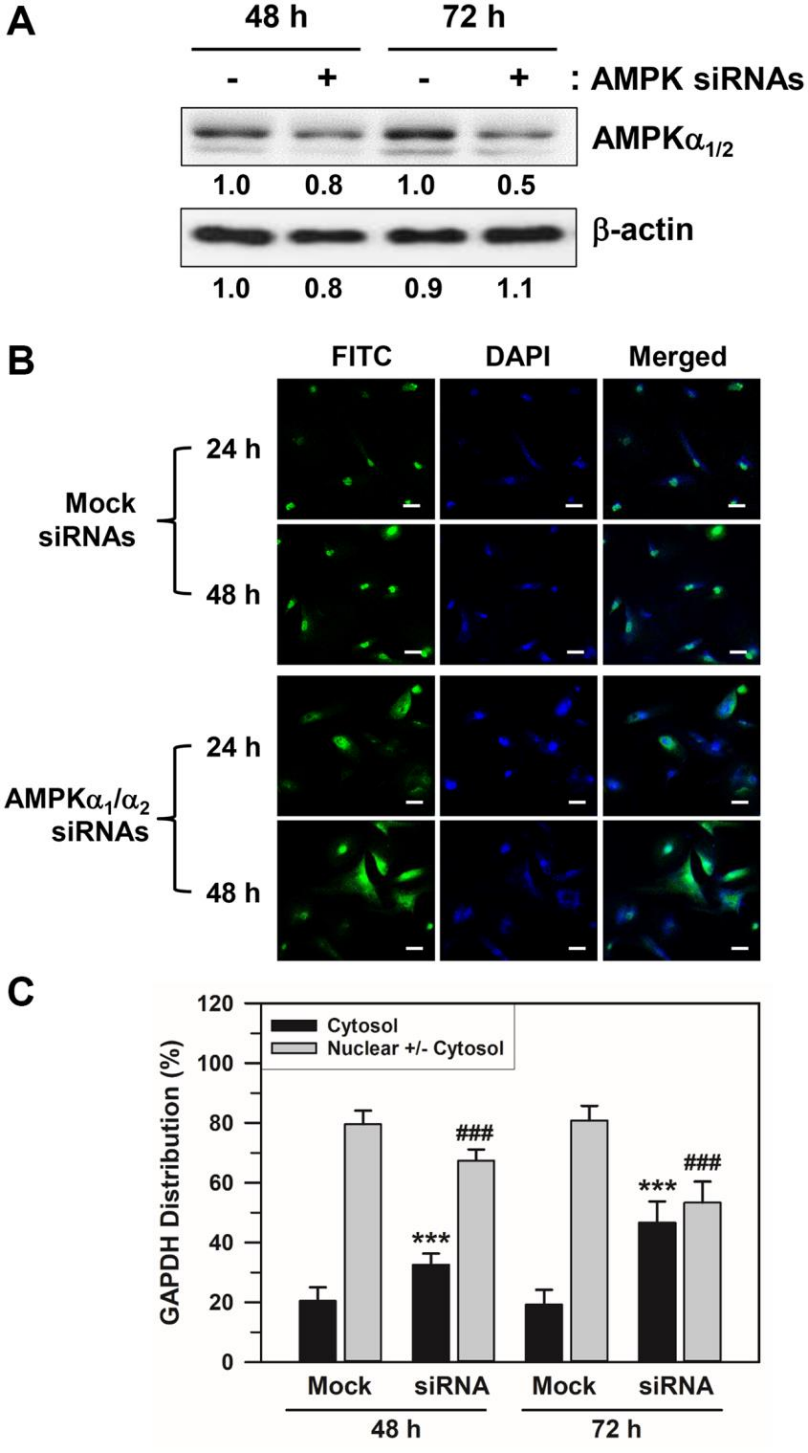
**Effects of AMPKα siRNA transfection on the nuclear accumulation of GAPDH in senescent HDFs.** Senescent HDFs were transfected with control scrambled siRNA duplexes (−) or siRNAs against AMPKα1/2 (+) for 48 and 72 h in DMEM with 10% FBS. (**A**) Cells were harvested at the indicated times after transfection, and lysates containing the same amount of protein (45 μg) were assessed by western blot analysis using polyclonal anti-AMPKα1/2 and anti-β-actin antibodies. The band densities were normalized against β-actin and the fold changes of AMPKα1/2 in AMPKα siRNA-transfected cells (+) compared to that of scrambled siRNA-treated control cells (−) are written under each band. (**B**) Cells were immunostained against GAPDH and analyzed by confocal laser scanning microscopy (magnification, 100×; scale bar, 50 μm). (**C**) The number of cells with cytosolic GAPDH alone (Cytosol) and cells having nuclear GAPDH with or without cytosolic GAPDH (Nuclear +/− Cytosol) was counted, and the percentage distributions were calculated (*n* = 40 for total replicates) and plotted as means ± standard deviations. ^***^p < 0.001 (Cytosol) and ^###^*p* < 0.001 (Nuclear +/− Cytosol), compared with the mock control siRNA.

We applied an AMPKα-specific siRNA approach to support the effect of AMPKα isoforms on the nuclear accumulation of GAPDH in senescent HDFs. Because the major isoforms of the AMPKα subunit in HDFs are AMPKα_1_ and AMPKα_2_, we used a mixture of siRNAs against AMPKα_1_ and AMPKα_2_ to block the expression of both isoforms. As depicted in Figure 5A and Supplementary Figure 3, AMPKα siRNA transfection for 48–72 h reduced the total AMPKα_1/2_ protein expression. Moreover, AMPKα siRNA-transfected HDFs presented with increased amounts of cytosolic GAPDH when compared with the control scrambled siRNA duplex-transfected HDFs (Figure 5B). AMPKα_1/2_ depletion by siRNA treatment significantly prevented the nuclear accumulation of GAPDH compared to the mock siRNA transfection group. Statistically significant differences between groups were determined by one-way ANOVA (*F*(3,156) = 239.297, *p* < 10^–3^) followed by Dunnett’s T3 post hoc test. The percentage distributions of HDFs with cytosolic GAPDH only for the mock control group were 20.44 and 19.21% at 48 and 72 h, respectively. In the AMPKα siRNA-transfected cells, these were increased significantly (^***^p < 0.001) to 32.55 and 46.68% at 48 and 72 h, respectively. In contrast, the percentage distribution of HDFs with nuclear GAPDH decreased from 79.56 to 67.45% at 48 h and from 80.79 to 53.32% at 72 h (^###^*p* < 0.001). These data support the idea that AMPK activation is responsible for the nuclear accumulation of GAPDH in senescent HDFs.

### AICAR- and SFM-induced GAPDH translocation in AMPKα_1_/α_2_ knockout mouse embryonic fibroblasts MEFs

**Figure 6:**
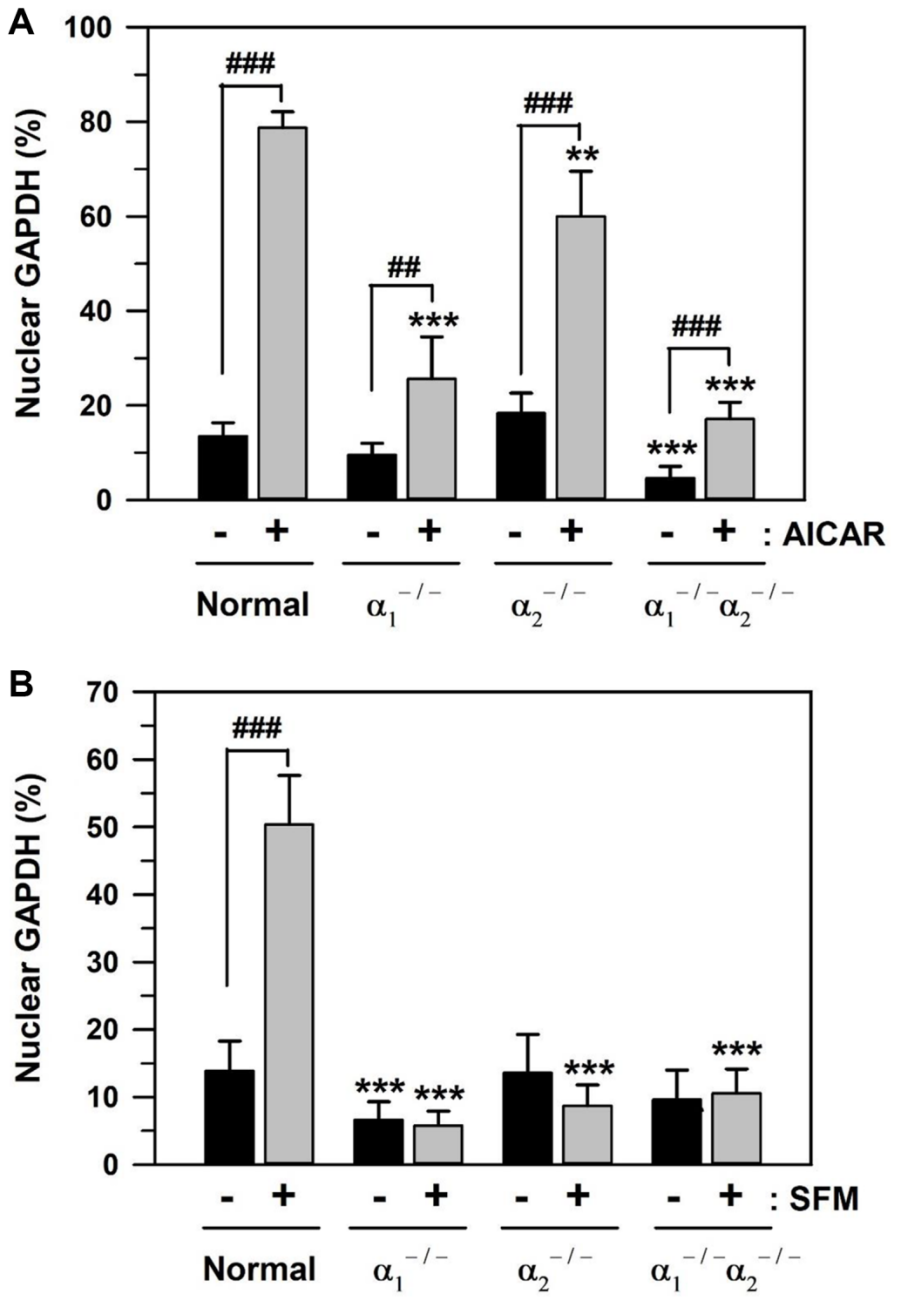
**AICAR- and SFM-induced GAPDH translocation in AMPKα-knockout MEFs.** (**A** and **B**) Normal and AMPKα-null MEF cells (AMPKα1^−/−^, AMPKα2^−/−^, and AMPKα1^−/−^/α2^−/−^) were cultured in 10% FBS medium for 2 days and then treated with 1 mM AICAR (**A**) or SFM (**B**) for 2 days. Cells were immunostained with a GAPDH-specific antibody and analyzed by confocal laser scanning microscopy. The number of cells having nuclear GAPDH with or without cytosolic GAPDH was counted, and the percentage distributions were calculated (*n* = 10 and 20 for total replicates in A and B, respectively) and plotted as means +/− standard deviations. ^**^p < 0.01 and ^***^p < 0.001, compared with the normal value in the absence of AICAR or SFM (−), ^##^*p* < 0.01 and ^###^*p* < 0.001, compared each value in the presence (+) to that in the absence of AICAR or SFM (−).

Using three types of AMPKα-knockout MEFs, AMPKα_1_ null (α_1_^−/−^), AMPKα_2_ null (α_2_^−/−^), and AMPKα_1_ and α_2_ null (α_1_^−/−^α_2_^−/−^), the effects of the catalytic α subunit of AMPK on GAPDH translocation were assessed. The baseline level of nuclear GAPDH and the level of its nuclear translocation upon addition of 1 mM AICAR or culturing in SFM for 48 h were compared in control MEFs and AMPKα-knockout MEFs. The statistically significant difference between groups was determined by one-way ANOVA (*F*(7,72) = 242.501, *p* < 10^–3^) followed by Dunnett’s T3 post hoc test. The control MEFs showed a significant increase in the level of nuclear GAPDH upon AICAR addition to 78.74% (5.84-fold increase), compared to the basal level of 13.49% (Figure 6A). Compared to the control MEFs, the baseline levels of nuclear GAPDH were reduced to 9.53 and 4.63% (^***^p < 0.001) in AMPKα_1_^−/−^ and AMPKα_1_^−/−^/α_2_^−/−^ MEFs, respectively. The rates of nuclear GAPDH upon AICAR addition significantly (^###^*p* < 0.001) decreased to 25.65, 60.06, and 17.15% in AMPKα_1_^−/−^, AMPKα_2_^−/−^, and AMPKα_1_^−/−^/α_2_^−/−^ MEFs, respectively, compared to 78.74% in control MEFs. These data indicate the critical role of AMPKα_1_ and the partial role of AMPKα_2_ in the nuclear translocation of GAPDH in MEFs.

Similar to AICAR, the effect of serum depletion was also examined in the control and three types of AMPKα-knockout MEFs. The statistically significant difference between groups in Figure 6B was determined by one-way ANOVA (*F*(7,152) = 217.107, *p* < 10^–3^) followed by Dunnett’s T3 post hoc test. Culturing in SFM for 48 h significantly (^###^*p* < 0.001) induced nuclear GAPDH from the baseline level of 13.86 to 50.38% (3.63-fold increase) in control MEFs (Figure 6B). In AMPKα_1_^−/−^ MEFs, the baseline levels of nuclear GAPDH were significantly reduced to 6.65% (^***^p < 0.001), compared to 13.86% of the control MEFs. Furthermore, the levels of nuclear GAPDH in SFM culture significantly (^***^p < 0.001) decreased to 5.78, 8.73, and 10.55% in AMPKα_1_^−/−^, AMPKα_2_^−/−^, and AMPKα_1_^−/−^/α_2_^−/−^ MEFs, respectively, compared to 50.38% in control MEFs (Figure 6B). These results suggest that both AMPKα_1_ and AMPKα_2_ proteins are necessary for SFM-induced GAPDH nuclear translocation.

## DISCUSSION

The intracellular localization of GAPDH is closely related to the diverse functions that GAPDH performs. Particularly, nuclear translocation of GAPDH is necessary for the transcriptional regulation of gene expression, DNA repair, and apoptosis [12–15], and cellular senescence via telomere shortening [16]. Because GAPDH was shown to translocate to the nucleus under various stress conditions, such as serum depletion [50] and oxidative stress [22], aging-associated growth signal deficits and oxidative stress may be involved in GAPDH nuclear translocation. Our present study indicated that GAPDH is distributed mainly in the nucleus in senescent HDFs, compared to young HDFs, and rat back skin cells (Figure 1). Permanent nuclear localization of GAPDH might correlate well with senescent-like growth arrest.

Nuclear localization of GAPDH can be modulated by cellular signaling cascades, including phosphoinositide 3-kinase (PI3K) and AMPK signaling pathways, in HDFs [50] and in pancreatic ductal adenocarcinoma [20]. Growth stimuli activate the PI3K signaling cascade, which leads to the nuclear export of GAPDH translocated by serum depletion, presumably via the activation of AMPK activity, in young HDFs. Therefore, we assumed that the nuclear accumulation of GAPDH in senescent HDFs could be reversed by the re-addition of serum or growth factors, such as PDGF and LPA. However, the age-related GAPDH nuclear deposits were reversed by treatment with LPA, but not PDGF (Figure 2B–2C). PDGF-induced signaling events are dramatically reduced, possibly due to the reduction in PDGF receptor expression, while a decrease in LPA-induced signaling events is less evident with aging [52, 53]. Li et al. also supported that as HDFs senesce, they become unresponsive to PDGF stimulation [54]. In contrast, LPA increases the proliferation of senescent HDFs by inhibiting AMPKα expression [55]. Hence, the nuclear export of GAPDH with the addition of LPA in our study could be a result of AMPK inhibition.

Furthermore, we found that age-related GAPDH translocation might be reversed by the addition of young ECM (Figure 3). This is consistent with a previous study that found morphologic rejuvenation of senescent cells with the addition of young ECM [9]. We conclude that age-related changes in the ECM include alterations in biochemical and/or biomechanical components that influence the upstream signaling pathway of GAPDH nuclear translocation. The assessment of the specific components involved in age-related changes was beyond the scope of the study and remains as an interesting aspect to explore further.

The dysregulation of ECM composition and structure contributes to several pathological conditions such as fibrosis and invasive cancer [56]. Fibrosis is a common process characterized by excessive ECM accumulation, whereas dynamic remodeling of the ECM around invasive cancer cells is implicated in tumor progression [57]. Accumulating evidence suggests that the PI3K/Akt signaling pathway plays a positive role in the pathological formation of fibrosis [58] and various human cancers [57, 59]. In contrast, AMPK alleviates fibrogenesis and ECM production in various organs and tissues [44]. Metformin-induced AMPK activation is indirectly associated with a lower incidence of pancreatic cancer in patients with type 2 diabetes [60]. Metformin inhibited the proliferation of pancreatic stellate cells in the pancreatic tumor stroma and decreased the production of ECM proteins by activating AMPK phosphorylation. As the PI3K/Akt pathway is involved in various biological processes, several on-target and off-target effects may be observed, which can limit the clinical development of therapeutics. AMPK activators such as metformin, AICAR, poricoic acid A, and HL156A may function as potential therapeutic agents for both fibrosis and cancer in aging organs [61–65].

Although AMPK activation is thought to delay aging [45], hyperactive AMPK has been linked to age-related diseases, such as Alzheimer’s disease and other cognitive dysfunctions [4, 49]. Therefore, further studies are needed to elucidate the role of AMPK in aging and age-related diseases. Previously, the AMP:ATP ratio and basal AMPK activity were found to increase during cellular senescence [66] in kidney cells and skin cells of aged rats [55, 67] and in the liver and brain hippocampus of aged mouse [4, 68]. In other studies, AMPKα phosphorylation and AMPKα_1_ and AMPKα_2_ activities were unaltered during aging at rest, but activation of AMPKα_1_ was enhanced, while activation of AMPKα_2_ was suppressed immediately after endurance-type muscle contractions in aged rats, resulting in inactive heterotrimer composition of AMPK and consequently increasing skeletal muscle atrophy sarcopenia [69]. In our study, we observed that basal AMPK activity was elevated in senescent HDFs, as indicated by enhanced phosphorylation on Thr^172^ (Figure 4A). Although the basal activity of AMPK is elevated or unaltered in a context-dependent manner, the responsiveness of AMPK signaling to cellular stresses and an acute or chronic activator of AMPK seems to decline during the aging process [46, 68, 70]. The molecular mechanism of age-related changes in AMPK activation capacity is poorly understood, but its dephosphorylation on Thr^172^ by phosphatases and inhibitory phosphorylation by kinases in the upstream signaling pathways might also play a role in the modulation of AMPK activation [34, 38].

In the present study, various methods of AMPK activity modification were employed to modulate GAPDH translocation in senescent cells: the AMPK activator AICAR increased GAPDH nuclear translocation, possibly because of higher levels of phosphorylated AMPK, but the AMPK inhibitor CompC induced GAPDH nuclear export (Figure 4C). Interestingly, at an IC_50_ value of 0.1–0.2 μM, CompC inhibited AMPK, but the expression of a few other protein kinases, such as ERK8 and PDGF receptor tyrosine kinase, were also inhibited with similar or greater potency [71, 72]. Moreover, the effect of CompC and its mechanism of action depend on the cell type and context [73, 74]; therefore, the validity of CompC as an AMPK blocker should be further assessed. Moreover, the PI3K/Akt pathway has been implicated in the regulation of nuclear translocation of GAPDH [50]. Because AICAR inhibits the PI3K/Akt signaling pathway in an AMPK-dependent manner [75] or AMPK-independent manner [76], AICAR may partly affect the PI3K and downstream Akt signaling pathways in a negative manner. Moreover, the addition of AICAR leads to AMPK activation along with Akt dephosphorylation, which suggests a coordinated inverse regulation of AMPK and Akt [77, 78]. Some growth signals or chemicals that activate the PI3K/Akt pathway could reverse the senescence-associated nuclear localization of GAPDH, presumably rejuvenating senescent cells. LPA and young ECM may stimulate mitogenic signals, such as PI3K and Akt, to a limited degree in senescent cells [52, 53]. Therefore, future studies of the molecular mechanisms underlying GAPDH nuclear translocation besides via AMPK-dependent phosphorylation are necessary to understand this phenomenon.

The role of AMPK protein expression in GAPDH translocation was also confirmed by AMPKα_1_/α_2_ siRNA transfection and utilization of AMPKα-knockout MEFs, AMPKα_1_ null (α_1_^−/−^), AMPKα_2_ null (α_2_^−/−^), and AMPKα_1_/α_2_ null (α_1_^−/−^α_2_^−/−^). AMPKα depletion by AMPKα_1/2_ siRNA transfection reduced AMPKα_1/2_ protein levels (Figure 5A) and reduced nuclear accumulation of GAPDH in senescent HDFs (Figure 5B, 5C). Although the basal levels of nuclear GAPDH were changed differently in AMPKα_1_- and AMPKα_2_-knockout MEFs, the levels of nuclear GAPDH translocated upon AICAR and SFM addition were reduced in both AMPKα_1_-knockout and AMPKα_2_-knockout MEFs (Figure 6A and 6B, respectively). These results suggest that both AMPKα_1_ and AMPKα_2_ proteins are necessary for AICAR- and SFM-induced GAPDH nuclear translocation.

At present, the role of the AMPK-dependent translocation of GAPDH in senescent cells has not been elucidated. The effect of AMPK activation differs depending on the type of cell or tissue and the type and duration of the damaging agent. AMPK activation and AICAR treatment significantly inhibited the proliferation of various cell lines and cancer cells via cell cycle arrest, accompanied by the increased expression of p21, p27, and p53 proteins and inhibition of the PI3K/Akt pathway [79]. Conversely, the proliferation potential of senescent HDFs can be increased by inhibiting the AMPK signaling pathway [55]. AMPK also contributes to UV- and H_2_O_2_-induced apoptosis in human skin keratinocytes by inhibiting mTOR and positively regulating p53 and p38 expression [80]. AMPK activation by AICAR treatment induces apoptosis in B-cell chronic lymphocytic leukemia cells [81], retinoblastoma cells [82], and rat pituitary tumor cells [83]. Moreover, AMPK is required for the pro-apoptotic effects of quercetin in non-malignant cells as well as in various tumor cells [84]. In contrast, the AMPK pathway plays a protective role against insult-induced apoptosis. For example, AMPK activation reduces anoxia-induced apoptosis [85] and high glucose-induced apoptosis in HUVECs [86], presumably through the regulation of autophagy [36]. AMPK activation is also an essential component of the adaptive response to cardiomyocyte stress that occurs during myocardial ischemia [47]. Therefore, pharmacological activation of AMPK can be applied as a cardioprotective strategy for the treatment of myocardial infarction [48]. Depending on the presence of downstream targets of AMPK, such as mTOR, SIRT1, Nrf2, NF-κB, PI3K/Akt, and p38 MAPK, AMPK can exert opposite functions in different cells.

The effect of nuclear GAPDH also depends on the type of cell and insult. Nuclear GAPDH induces apoptosis triggered by various cytotoxic stressors [87–90]. The mechanism underlying GAPDH translocation and subsequent cell death is related to several protein factors such as SIRT1, p53, Bcl-2, and Siah1 [90]. Mutant p53, predominantly found in various aggressive cancer cells, prevents GAPDH translocation, supports glycolysis in cancer cell growth, and inhibits cell death mediated by nuclear GAPDH [20]. Phosphorylation of GAPDH at Ser122 by AMPK, S-nitrosylation, S-glutathionylation, acetylation, and Siah1 binding to GAPDH are required for the nuclear translocation of the enzyme [19, 23, 26, 27]. Phosphorylation by Src and Akt2, SIRT1 expression by mutant p53, and subsequent deacetylation by SIRT1 in the cytoplasm prevent GAPDH translocation [24]. Under oxidative conditions, oxidized GAPDH is translocated to the nucleus to form aggregates, which act as a seed to accelerate amyloidogenesis, resulting in apoptosis and neurodegenerative diseases [22, 28]. In contrast, nuclear GAPDH also exhibits cytoprotective effects by participating in various cellular events such as autophagy and DNA repair [14, 21]. Whatever way they work, AMPK-dependent nuclear translocation of GAPDH seems to play an important role in the fate of senescent cells and in age-related diseases.

**Figure 7:**
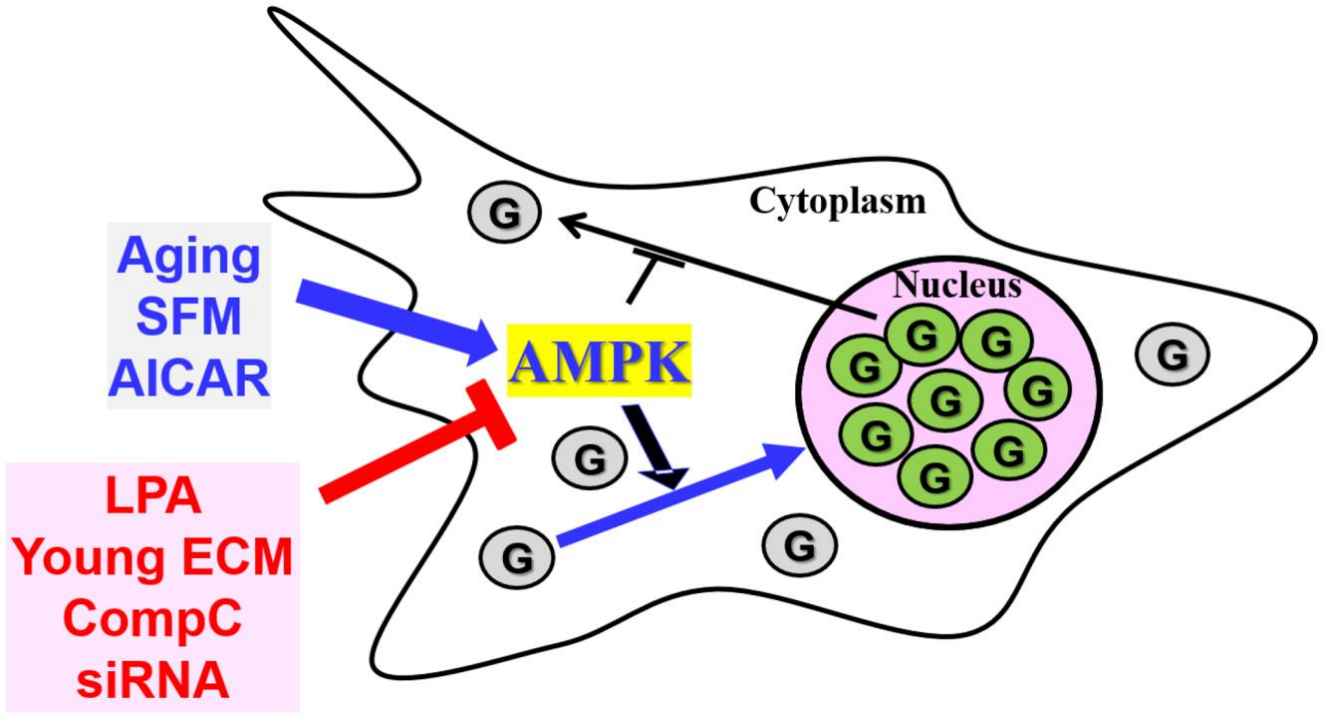
**The AMPK signal transduction pathway is involved in the nuclear translocation of GAPDH in senescent HDFs.** GAPDH (G) is present mainly in the nucleus of senescent HDFs and other cells from aged animals. When senescent cells are serum-depleted by incubation with SFM or treated with the AMPK activator AICAR, which may result in AMPK activation, GAPDH translocates completely to the nucleus. AMPK inhibition by LPA, young ECM, CompC and AMPKα-siRNAs prevents basal and SFM- or AICAR-induced nuclear translocation of GAPDH. Taken together, the nuclear accumulation of GAPDH in senescent cells can be altered by the modulation of AMPKα expression and activation.

Taken together, our results, summarized in Figure 7, suggest that the nuclear accumulation of GAPDH might be due to enhanced AMPK activity in senescent HDFs, which could possibly be reversed with proper modification of AMPK activity.

## MATERIALS AND METHODS

### Materials

PDGF-BB, LPA, fluorescein isothiocyanate (FITC)-conjugated goat anti-mouse secondary antibody (#F0257), and mouse anti-β-actin monoclonal antibody (#A5441) were purchased from Sigma-Aldrich (St. Louis, MO, USA). Dulbecco’s modified Eagle’s medium (DMEM), FBS, opti-MEM I, penicillin, and streptomycin were obtained from Gibco/BRL Life Technologies, Inc (Carlsbad, CA, USA). Rabbit monoclonal antibodies against AMPKα (#2532S) and phospho-Thr^172^-AMPKα (40H9: #2535S), and rabbit monoclonal anti-GAPDH IgG (#2118S) for immunohistochemistry were purchased from Cell Signaling Technology (Denver, MA, USA). CompC and AICAR were purchased from Calbiochem (San Diego, CA, USA) and mouse anti-GAPDH monoclonal antibody (#MAB374) was from Chemicon (Bedford, MA). Horseradish peroxidase-conjugated anti-rabbit (#PI-1000) and anti-mouse (#PI-2000) secondary antibodies were obtained from Vector Laboratories (Burlingame, CA, USA) and protein assay kit was from Bio-Rad Laboratories (Hercules, CA, USA). Trypsin and Zymed Picture Plus Kit was purchased from Zymed (South San Francisco, CA, USA), and predesigned human AMPKα_1/2_-specific and control scrambled siRNA duplexes were purchased from Santa Cruz Biotechnology (Santa Cruz, CA, USA). Lipofectamine^™^, RNAiMAX, and Permount mounting solution including 4′,6-diamidino-2-phenylindole (DAPI) were obtained from Invitrogen Life Technologies (Carlsbad, CA, USA) and enhanced chemiluminescence (ECL) system was from GE Healthcare (Buckinghamshire, UK, USA).

### Cell culture

HDFs were isolated from the foreskin of neonates, as described previously [91]. HDFs were cultured in DMEM supplemented with 10% FBS, 100 U/mL penicillin, and 100 μg/mL streptomycin in 100 mm tissue culture dishes and were maintained in a humidified 5% CO_2_ incubator at 37°C. The cells were divided into three groups depending on the duration of growth: young, middle, and senescent. Young HDFs were cultured until PD was less than 25, middle HDFs until PD between 45 and 65, and senescent HDFs until PD greater than 65. Senescent HDFs were identified by their characteristic morphological changes, enhanced beta-galactosidase activity, and cell growth arrest. Before serum depletion, regardless of their classification, HDFs were grown for 2 days to reach 60–70% confluency in a culture medium containing 10% FBS. To determine the effect of serum or growth factor re-addition on the nuclear localization of GAPDH, subconfluent senescent HDFs were serum-deprived via a 5-day-incubation in SFM containing 0.1% bovine serum albumin (BSA).

### Immunofluorescence staining

For immunofluorescence staining, 1.0 × 10^5^ HDFs were cultured on coverslips in 6-well culture plates for 1 day and treated with the indicated medium or reagents for the indicated times. The cells on coverslips were washed twice with ice-cold phosphate-buffered saline (PBS), fixed with 4% paraformaldehyde in PBS for 10 min, and washed with PBS. Subsequently, blocking solution with 2% BSA in Tris-buffered saline with Tween 20 (TBST: 20 mM Tris, 138 mM NaCl, 0.1% Tween 20, pH 7.4) was used to saturate non-specific protein binding sites for 1 h with gentle shaking. The cells were incubated with primary monoclonal anti-GAPDH antibody (1:500~1,000 in blocking solution) for 1 h at 25°C, and were washed three times with ice-cold TBST for 10 min each. Cells were incubated with FITC-conjugated anti-mouse secondary antibody (1:500) in blocking solution for 30 min. Cells were washed three times with TBST for 10 min each, and were dehydrated in three washes with 100% methanol for 1 h. The coverslips were mounted on glass slides with a mounting solution containing DAPI for nuclear staining, and fluorescence images were captured with a confocal laser scanning microscope (FV500; Olympus, Tokyo, Japan). GAPDH was indicated by FITC fluorescence, which glows in green. Blue DAPI-stained nuclei and merged images are also visible in confocal microscopic pictures. Each sample was counted in 10–20 different fields at 100× magnification. The data were combined to calculate the total cell number of approximately 100 and were converted to percentage distribution. All experiments were repeated at least three times to obtain reliable statistical inferences.

### Transfection of human AMPKα siRNAs

Senescent HDFs were transfected with 50 nM human AMPKα_1/2_-specific or control scrambled siRNA duplexes, as described previously [50]. Briefly, 2.0 × 10^4^ senescent HDFs per well were seeded in 6-well plates in 2 mL of antibiotic-free culture medium supplemented with FBS. Cells were incubated at 37°C in a CO_2_ incubator overnight until the cells were 30–50% confluent. The mixture of 50 pmol siRNA duplex in 100 μL of Opti-MEM I reduced serum medium and 2 μL of Lipofectamine^™^ RNAiMAX transfection reagent were preincubated at room temperature for 15 min and then diluted in 1 mL of transfection medium. The mixtures were overlaid onto the washed cells. After 6 h of transfection at 37°C in a CO_2_ incubator, 1 ml of DMEM containing 20% FBS and 2× antibiotics was added to each well, and cells were incubated for an additional 24 h at 37°C. After refreshing the culture medium to DMEM containing 10% FBS and 1× antibiotics, cells were incubated for 48–72 h and assayed for AMPKα_1/2_ levels using western blot analysis and for GAPDH localization using immunofluorescence staining and confocal microscopy.

### Western blot analysis

Protein expression levels were examined by western blot analysis, as described previously [72]. A total of 1 × 10^6^ HDFs grown in 100-mm culture dish were lysed in lysis buffer (50 mM Tris-HCl, pH 7.5, 150 mM NaCl, 2 mM EDTA, 1 mM EGTA, 1 mM Na_3_VO_4_, 10 mM NaF, 1 mM DTT, 1 mM PMSF, 25 μg/mL leupeptin, 25 μg/mL aprotinin, 5 mM benzamidine, and 1% Ingepal CA630) at 4°C for 30 min on a rocker. The lysates were centrifuged at 14,000 × *g* for 15 min in an Eppendorf centrifuge to remove cell debris, and the protein concentration of each lysate was determined using a Bio-Rad protein assay kit, according to the manufacturer’s protocol. Cell lysates of equal amounts of protein were resolved by sodium dodecyl sulfate-polyacrylamide gel electrophoresis (SDS-PAGE), and transferred electrophoretically onto Immobilon PVDF membranes. A solution containing 5% non-fat dried milk or 1% BSA in TBST buffer was used to block the blots for 1 h at 25°C. Moreover, proteins were incubated overnight with monoclonal antibody (1:200 to 1: 1000) in the blocking solution at 4°C, washed three times with TBST, and further probed with horseradish peroxidase-conjugated anti-rabbit secondary IgG (1:5000) for 30 min. An ECL detection system was used to visualize the immune complexes on the blots.

### Rat skin section preparation and immunohistochemistry

Adult male Fisher 344 rats were purchased from Samtaco BioKorea (Seoul, Korea). The animal study was approved by the Institutional Animal Care and Use Committee (IACUC) of the Korea Basic Science Institute (KBSI) (KBSI-ACE1913). The animals were housed two per cage in a room with controlled temperature and humidity with a 12-h light/12-h dark cycle. They were maintained on a standard diet with food and water ad libitum in an animal facility approved by the Korean Association for Assessment and Accreditation of Laboratory Animal Care. The back skin sections of young (6 months) and aged (24 months) rats were obtained as described previously [92]. Rats were anesthetized with an intraperitoneal injection of ketamine and xylazine (87/13 mg/kg). The back was shaved, and a 5-mm-thick skin section was cut. The sections were fixed overnight in ice-cold 10% paraformaldehyde and embedded in paraffin. Serial paraffin sections on the microslides were deparaffinized in xylene and rehydrated sequentially with ethanol solutions of different grades. Antigens were retrieved by incubation with 10 mM citrate buffer (pH 6.0) for 15 min in a 700 W microwave. Slides were treated with 3% hydrogen peroxide to inhibit endogenous peroxidase activity and were washed with 0.05 M Tris-buffered saline (pH 7.6). The slides were incubated in 5% skim milk for 1 h at 25°C to block nonspecific binding sites and then incubated overnight with primary rabbit monoclonal anti-GAPDH antibodies (1:500) at 4°C. The avidin-biotin peroxidase complex method was used to visualize the immune complex, which was counterstained with hematoxylin and eosin (H&E). After sequential dehydration using ethanol and xylene, the slides were mounted using Permount. The slides were photographed using a light microscope (×200). Each experiment was performed three times.

### Statistical analysis

For statistical analyses of the data, one-way analysis of variance (ANOVA) followed by Dunnett T3 post hoc test (SPSS, IBM, Seoul, Korea) was performed to assess significant differences between groups. Data are presented as the mean ± standard deviation of at least three experiments. *P* values of less than 0.05 were considered statistically significant.

## Supplementary Materials

Supplementary Figures
