# Supplementary material for: AMP-activated protein kinase-dependent nuclear localization of glyceraldehyde 3-phosphate dehydrogenase in senescent human diploid fibroblasts
Source: Aging (Albany NY). 2022 Jan 12;14(1):4–27. doi: 10.18632/aging.203825 (PMC8791203; doi:10.18632/aging.203825)
Supplement: Supplementary Figures [file aging-14-203825-s001.pdf]

SUPPLEMENTARY FIGURES

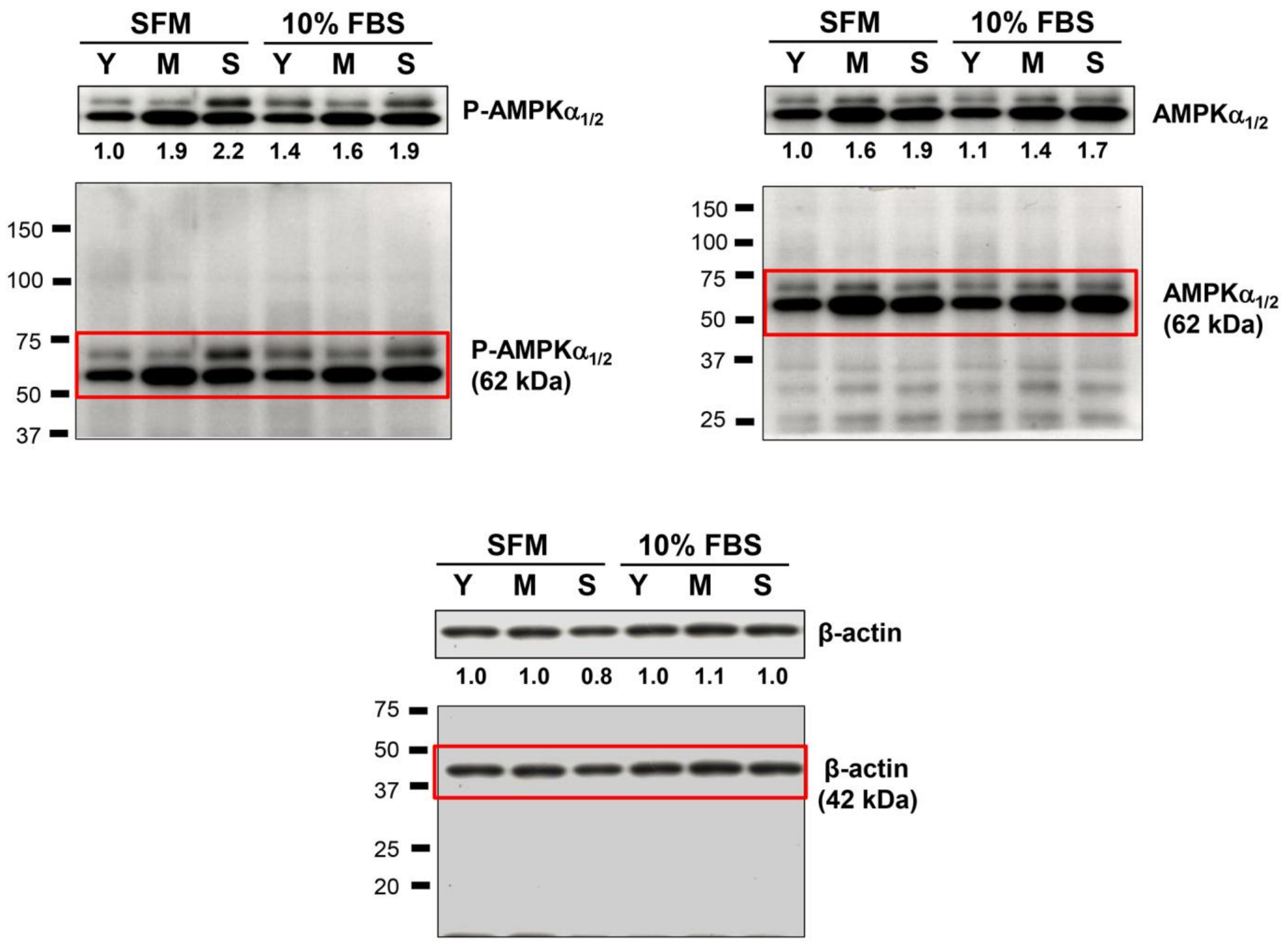

Supplementary Figure 1. A representative whole blot for western blot analysis of each protein in Figure 4A. Subconfluent young (Y, PD 12), middle (M, PD 48), and senescent (S, PD 86) HDFs were incubated with SFM or 10% FBS medium for 2 days. Cells were lysed in a lysis buffer, and 45 µg of protein from each lysate was assessed for the levels of phosphorylated $AMPK\alpha_{1/2}$ on Thr^{172} (P-$AMPK\alpha_{1/2}$), total $AMPK\alpha_{1/2}$, and β-actin by western blot analysis. The band densities were normalized against β-actin and the fold changes compared to that of young cells (Y/SFM) are written under each band. A representative whole blot for western blot analysis of each protein are shown below each band.

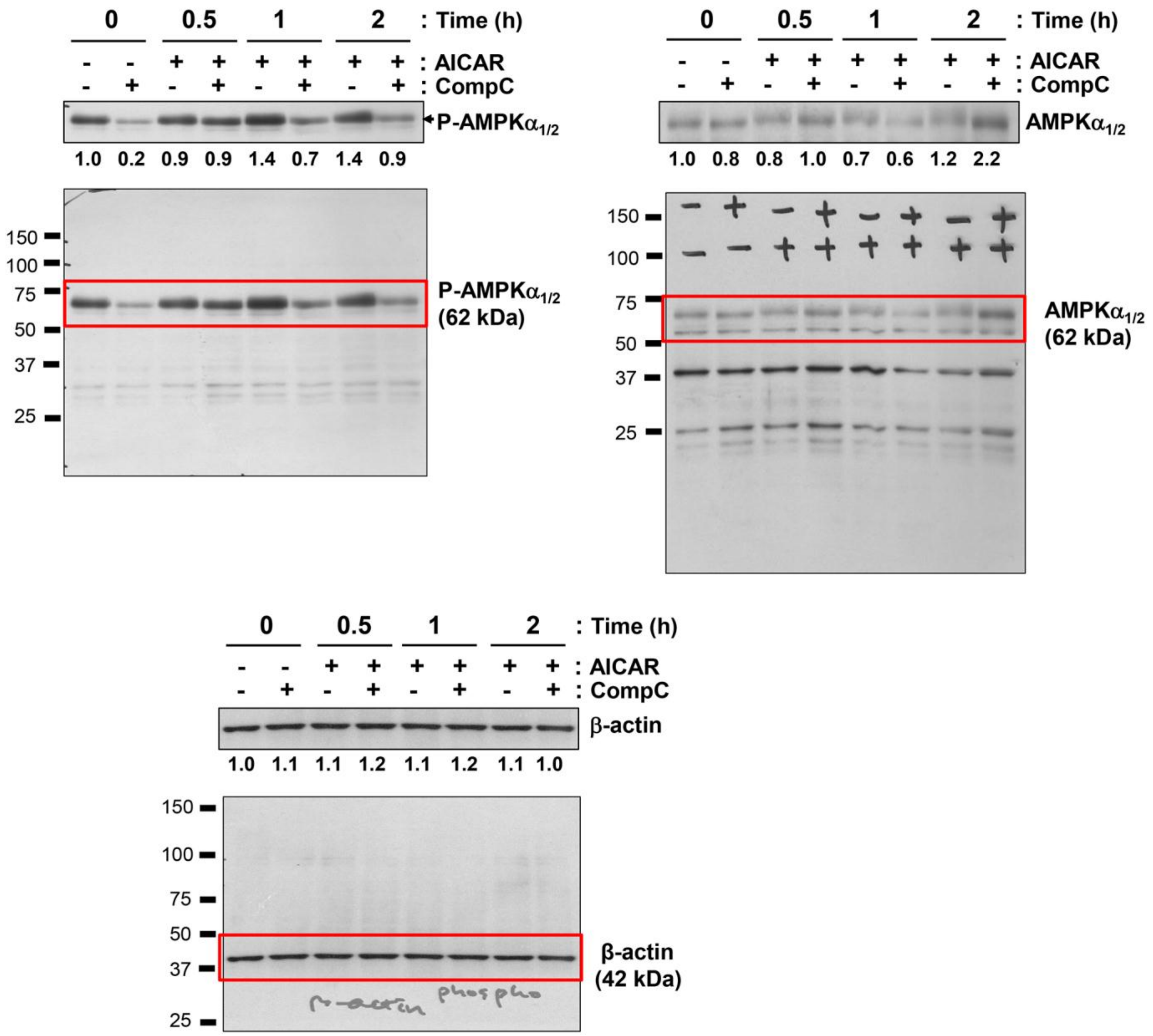

Supplementary Figure 2. A representative whole blot for western blot analysis of each protein in Figure 4B. Subconfluent young (PD 20) and senescent (PD 74) HDFs were treated with vehicle (–) or 1 mM AICAR and/or 10 µM CompC (+) for 2 days. Cells in A and B were lysed in a lysis buffer, and 45 µg of protein from each lysate was assessed for the levels of phosphorylated AMPK$\alpha_{1/2}$ on Thr^{172} (P-AMPK$\alpha_{1/2}$), total AMPK$\alpha_{1/2}$, and β-actin by western blot analysis. The band densities were normalized against β-actin and the fold changes compared to that of vehicle treated control cells (–/–) are written under each band. A representative whole blot for western blot analysis of each protein are shown below each band.

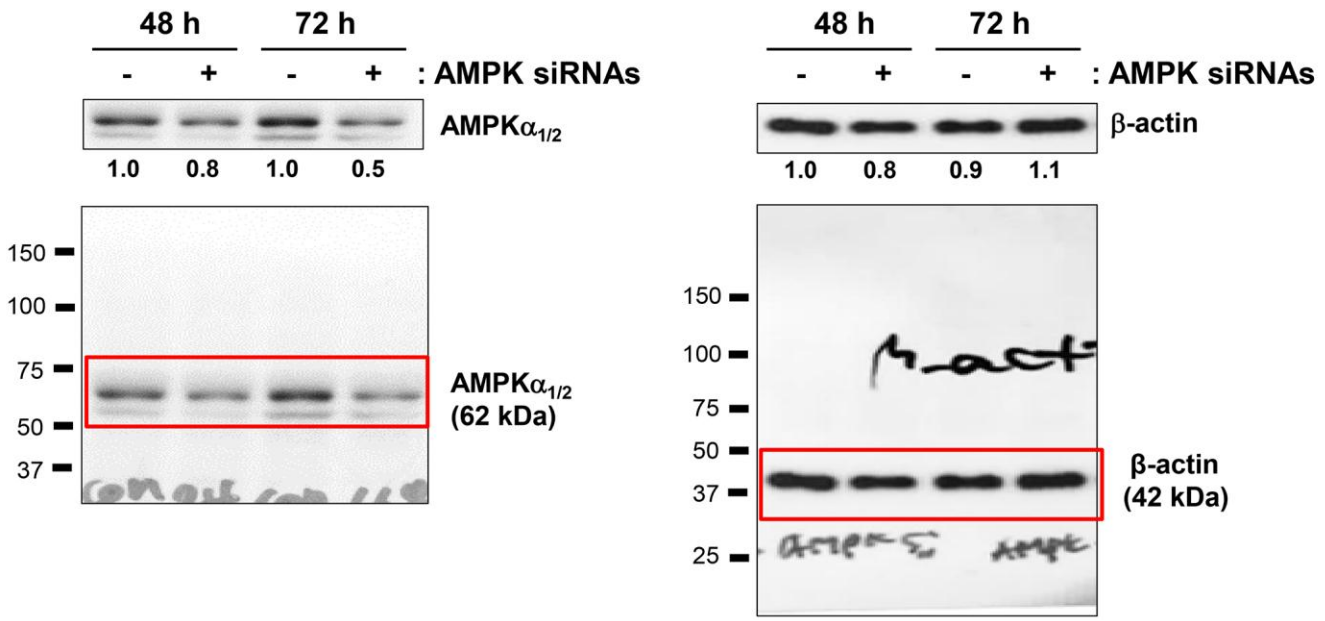

Supplementary Figure 3. A representative whole blot for western blot analysis of each protein in Figure 5A. Senescent HDFs were transfected with control scrambled siRNA duplexes (–) or siRNAs against AMPKα$_{1/2}$ (+) for 48 and 72 h in DMEM with 10% FBS. Cells were harvested at the indicated times after transfection, and lysates containing the same amount of protein (45 μg) were assessed by western blot analysis using polyclonal anti-AMPKα$_{1/2}$ and anti-β-actin antibodies. The band densities were normalized against β-actin and the fold changes of AMPKα$_{1/2}$ in AMPK siRNA-transfected cells (+) compared to that of scrambled siRNA-treated control cells (–) are written under each band. A representative whole blot for western blot analysis of each protein are shown below each band.
